# Experiences and Perceptions of Police Officers Concerning Their Interactions With People With Serious Mental Disorders for Compulsory Treatment

**DOI:** 10.3389/fpsyt.2019.00187

**Published:** 2019-04-18

**Authors:** Ruben Soares, Mariana Pinto da Costa

**Affiliations:** ^1^Institute of Biomedical Sciences Abel Salazar, University of Porto, Porto, Portugal; ^2^Hospital de Magalhães Lemos, Porto, Portugal; ^3^Unit for Social and Community Psychiatry (WHO Collaborating Centre for Mental Health Services Development), Queen Mary University of London, London, United Kingdom

**Keywords:** law, experience, mental illness, police officers, coercion, compulsory, treatment, Portugal

## Abstract

**Background:** In Portuguese law, police officers are the link between security and the treatment of people with serious mental disorders who require compulsory admission. The perceptions of police officers are in part based on their individual characteristics, and may influence their capability in managing patients they are transporting. However, little is known about police officers experience of this process.

**Methods:** In-depth semi-structured interviews explored the experiences and per- ceptions of police officers from Porto Police Department in Portugal. All interviews were audio recorded, transcribed and analyzed through thematic analysis.

**Results:** Ten police officers agreed to take part in this study. The interviewed police officers consisted of nine men and one woman, had an average length service of 22.6 years and all had more than 10 years of service. The interviews highlighted that the activity of the police under the Mental Health Law is shaped by whether the person who they are transporting has a mental health disorder and requires psychiatric admission. The police officers reportedly adjusted their behavior to give patients more attention, comfort and empathy. However, they describe these interactions as one of the most time consuming and challenging activities for the police. Importantly, they acknowledged family members as crucial for police officers to be able to gain direct access to patients and knowledge about them. Police officers showed to perceive people with mental illness as unpredictable, dangerous and without discernment, and identified some aspects of the process that could be improved, such as hospital admission waiting times. Police officers felt they required more skilled support to deal with unwell patients.

**Conclusions:** This study highlights the perceptions and experiences of police officers about the process of compulsory admission, and identifies areas of unmet needs. These findings help to raise awareness of their needs, improving this process, and ensuring a more humane and effective approach.

## Introduction

Compulsory admission and treatment is a topic of intense debate in psychiatry. This is due to the effects such policies have on people's lives, given that it involves detaining them against their wishes and restricting their individual liberty (1). Compulsory treatment is ethically challenging, often associated with fear, exclusion and loss of self-determination (2, 3). The process is based on the assumption that the person is incapable of recognizing that their mental health has deteriorated and there is an urgent need for adequate treatment (4). The majority of mental health professionals disapproves coercive medical measures, but recognizes the impact upon patients' adherence to treatment (5). Some research has also found that a majority of patients understand that they were acutely unwell at the time of their admission and that coercive interventions enabled their recovery (6). However, some patients perceive their admission as a negative experience (6, 7), entailing an intrusion into their physical integrity and their liberty (3). These diverse views raise awareness to patients' potential feelings of ambivalence toward compulsory admissions.

Police officers are one of the first professionals to interact with people requiring compulsory admission due to their mental health. Research in North America (6, 8–12) and European countries (13), has highlighted that the approach adopted by the police officers is crucial to patient's cooperation (10, 14). Police officers' experience and personal characteristics play an important role in their own capacity in doing this task (15). Police officers with a higher level of education (12 years of education or more) appear to perceive people with mental disorders as less unpredictable and dangerous, and capable of having a family (16). Equally, police officers who performed more than six compulsory admission transportations used less physical force during them (16). Thus, the level of education and experience that police officers have in dealing with people with mental illness influences their behavior and perceptions about them (17). For that reason, education and contact should be effective strategies for changing police officers perceptions and attitudes toward people with serious mental disorders (18, 19). However, some police officers report that they do not feel properly trained to deal with a person with a serious mental disorder (9, 14), feeling unable to identify symptoms of mental illness or deal with psychotic or hostile behavior (6, 11).

Police officers see value in obtaining training to avoid potential bad practices (17). However, as a result of their lack of training some encounters between police officers and people with serious mental disorders result in abuse of force, precipitation of violent acts and sometimes even death (15, 20, 21). In the United States in 2015 about 23% (251 deaths) of the total number of deaths (*n* = 1099) resulted from police interactions with people with mental illness (22). The complexity of these interactions magnified the need for implementing standardized educational and training programs, as well as special police teams selected, trained and motivated to help people with serious mental disorders in crises (10, 23). These teams have demonstrated less aggressive interactions with people in crises when compared to police officers who did not receive such training (24). This complex reality has reinforced the need of more assistance from other groups (e.g., special medical local authorities), and a faster access to mental health services during this process (16, 25).

The procedural justice approach is seen as the main pathway to promote police legitimacy, and thus citizens' cooperation (26). This approach is grounded in two components (27): (i) the quality of the decision-making procedures (i.e., citizens participation in the proceedings before the authority's decision, giving citizens a voice, as well as the neutrality of such decision), and (ii) the quality of the treatment (i.e., the dignity and respect in which people are treated, and if the authorities' motives are perceived as trustworthy) (26, 28). These two components emerge into four key constructs that shape police encounters: (i) voice, (ii) trustworthy motives, (iii) dignity and respect, and (iv) neutrality in the decision making (26).

In Portugal, the Mental Health Law (Law n. ° 36/98, July 24th, 1998) aims to establish the main principles of Portuguese mental health policies and rules the compulsory admission for people with serious mental disorders (29). Although the Fundamental Health Law (Law n. °. 48/90, August 24th, 1990) establishes the patient's rights and duties in the Portuguese services (30), the mental health patients are endowed by the Portuguese Mental Health Law with more rights and duties. This illustrates the legal recognition of the idiosyncratic nature of mental illness, and its repercussions on the self-determination capacities of the person with a serious mental disorder, as well as the specific implications of psychiatric treatment, which obligates to ensure other rights and duties. Thus, the Mental Health Law is a crucial and nuclear framework declaration of mental health rights in Portuguese legal order (31). Its principles are more connected to guardianship and protection rather than medical or social care. This means that the juridical model prevails over the therapeutic model (32), as the assessment of the legal status of people with mental illness is governed by constitutional principles and norms, leading by equality and non-discrimination (33).

The Portuguese Mental Health Law (on 23rd article, urgent admission) gives a special role to the police officers who are responsible for the transportation of people with serious mental disorders to the psychiatric emergency service closest to their residence (29), and are the link between the security and treatment of these patients. Between 1999 and 2007 the percentage of compulsory admissions in Portugal at the *Hospital de Magalhães Lemos*, Porto, rose significantly. In 1999, 1 year after the implementation of the law, compulsory admissions represented 1.5% of all the admissions, whereas in 2007 this percentage rose to 7.2%. The same tendency is observed in *Hospital Júlio de Matos*, Lisbon, where in 1999 the amount of compulsory admissions represented 5.53% of all admissions compared to 15.31% in 2007 (34). Of utmost importance is the article cited to begin the process, because this determines whether the police are involved or not (e.g., in non-urgent situations). Between 2008 and 2010, 93.2% of all compulsory admissions at these two hospitals occurred following the article of urgent admission (35).

It is therefore clear the importance of police officers in compulsory admissions and in the delivery of involuntary mental health treatment. However, their interactions with people with serious mental disorders under the compulsory treatment procedure has not been studied. The lack of knowledge in this area requires attention as it is of public interest and would arguably lead to improvements in the mental health care, to ensure it is delivered with dignity and justice.

## Methods

### Settings and Participants

The study was conducted in six police departments of PSP (Polícia de Segurança Pública), in Porto, which were selected as they were those with higher number and frequency of compulsory admissions, covering a broad geographical area of Porto metropolitan police department (Matosinhos, Ribeira, Santo Tirso, Maia, Foz, Lagarteiro), and thus giving us some meaningful insight into the topic of the study. An information sheet with information about the study was distributed to all police officers in the department. Further to that, the main researcher approached the police officers in each department, providing them with information about the researchers, the scope of the study, a brief description of the method, and the contact details of the main researcher for any further questions.

The only inclusion criteria were that police officers had completed at least one compulsory admission under the Mental Health Law.

### Data Collection and Analysis

A semi-structured interview topic guide was developed in consultation with a senior police officer (see Supplementary Material). The first author conducted in-depth semi-structured interviews exploring the experiences and perceptions of police officers of the compulsory admission process, and collected socio-demographic information (see Supplementary Material). All interviews were audio recorded using a digital voice recorder (Olympus WS- 853) and were transcribed verbatim. Contextual data relating to the approved police departments were also collected (e.g., a general description of the interview room like the climate conditions, natural light presence, and if there was any physical barrier between the interviewer and the interviewee).

Thematic analysis of the interview transcripts was conducted following the Braun and Clarke's (36) approach, and using the Nvivo 12 software. Police officers' names were replaced with numbers to protect their privacy. Initial codes were generated and then sorted into broader themes, with similar codes placed under the same theme. A theme was determined on the basis of significance to the research question. Themes were then revised and refined to ensure codes within each theme were greatly associated, and that each theme was distinctive. Themes were then named. Procedural consistency was guaranteed using a double-coding approach (by RS and MPC), with cross-checking between coders to ensure consistency.

## Results

Twelve police officers were approached, and ten agreed to take part. The police officers interviewed from Porto Police department consisted of nine males and one female, all caucasians, with a mean age of 46.4 years (age range from 34 to 58). The interviews ranged in duration from 25 to 57 min. All were ungraduated police officers, with an average length of service of 22.6 years and all had more than 10 years of service. None of them described having history of mental illness themselves. Although all departments did several compulsory admissions per year, the volume of compulsory admissions differed across police departments, ranging from one intervention per month to several, according to seasons (spring/autumn), festive dates, and vacations periods. All of them reported that they had no specific training to deal with people with serious mental disorders.

Five themes emerged from the analysis of the data: “*Police activity and compulsory admission*,” “*Family is core in the compulsory admission process*,” “*Triad of success*,” “*Views of mental illness*,” and “*Improvi*ng *the reality of compulsory admission.”* (Table 1).

**Table 1:** Themes and subthemes.

**Police activity and compulsory admission**	**Family is core in the compulsory admission process**	**Triad of success**	**Views of mental illness**	**Improving the reality of compulsory admission**
The importance of the law	Facilitate the access to the patient	Obtain knowledge about the patient	Cyclic	Mismatch between safety and health
The primacy of patients help	Source of information	Building a relationship	Beliefs about people with mental illness	Negative aspects resulting from the police intervention
The operational activity adjusted by the person's illness condition		To be patient and do not rush		Police officers difficulties
Police presence is what counts				Try other solutions
The instrumentalisation of the police				

### Police Activity and Compulsory Admission

Police activity inherent in compulsory admissions requires very specific characteristics of police officers. Police activity is ruled by a Mental Health Law which determines the exact form and range of their actions. Yet, they are required to deal with the behavior of a person that has not committed a crime, but who may require urgent care for their mental illness. Police officers are therefore placed in a very challenging situation where they have to balance the law, dealing sensitively with an acutely ill person and fulfilling their duty (Table 2).

**Table 2:** Police activity and compulsory admission quotes.

**Police activity and compulsory admission**	
The importance of the Law	“*From the moment we are compelling him to do something, in this case to go to the doctor…there has to be a law, a legality that allows us to do that. I think that this is the goal, to give legality to a procedure that will do well. But we have to give legality because we are restricting him of their freedom”* (police officer 03)
	“*If I would not have a mental health law, by which means would I deprive him of his freedom that amount of time that I have to take him to a hospital? Because we have that issue, about freedom deprivation. He is not detained, he is just deprived of his freedom during the moment that we force him to go to the hospital”* (police officer 01)
The primacy of patients help	“*These people, we have to understand that they are so.…in a state where they do not…they are not in their normal state and we have to try…we are in the first stage … we have to realize that we will deal with people who are not hmmm. They need our help and help from other professionals….”* (police officer 05)
	“*We have always to try to say that the reason why we are there is to help them. Therefore… using all the persuasion that we have to say: ‘we are here not to arrest you because you did not commit any crime. We are here just to help you…”'* (police officer 10)
The operational activity adjusted by the person's condition	“*He is a sick person. When I receive the warrant report they are referred as a patient ‘the patient this, the patient that,' so, if the warrant refers to them as patient I have to treat him as the patient that he is”* (police officer 01)
	“*If it is a person with a mental disorder, I automatically treat them with all dignity and respect, because if they were a normal person, some kind of attitudes I would not have” (police officer 09)*
	“*In order to watch over patients' privacy and not to create a negative image, ‘The police is there, what did that guy do?' Because the public will think that they did something. Therefore I do my interventions, in general, without my police uniform. […] We want to do these interventions with some discretion”* (police officer 09)
The presence of the Police is what counts	“* Maybe if the escort was made by the hospital security they would not respect him so much as they will do with a police officer”* (police officer 07)
	“*The patient knows that he cannot exceed himself. Because if he does, the police will be there to finish it. We already had some situations that the physician said ‘no sir I do not want the police here,' for me it is legit and makes sense, but few moments later they will call us because the patient has strangled his neck”* (police officer 03)
The instrumentalisation of the police	“*In situations that we have a warrant, our intervention is quite objective, we have to find the person or pick them up at home, transport them to the hospital, even against their will. Our role is fulfill and enforce.”* (police officer 03)

#### The Importance of the Law

The existence of a Law that regulates the compulsory admission is the cornerstone of the police activity. It gives them the ability and legitimacy to deprive someone's freedom in order to take them to a hospital to be seen by a doctor. Police officers in this study highlighted this legal legitimacy as a key factor, as in its absence they are unable to protect and help the person. Another important consideration for the police officers is they guarantee freedom “deprivation” rather than a “detention.” This entails the difference between a criminal detainee and a mentally ill person who has to be deprived of their freedom to seek medical support. This legal difference is well-recognized by the police officers and is enforced in their interventions.

#### The Primacy of Patients Help

The intervention of the police in mental health is shaped by the principle of helping others. In this case they are helping a person who suffers from a mental disorder and does not have the ability to recognize they are ill and need treatment. This is the starting point of the police approach to compulsory admission and is essential to a humanized interaction. Understanding mental illness and how it influences a person's cognitions and behaviors supports a more empathic interaction rather than what is seen as the typical authoritative police approach in which criminal people are detained. This understanding that a person's behavior is just the expression of their illness and a way to express the need of help shapes their whole operation and individual conduct.

#### The Operational Activity Adjusted by the Person's Condition

The existence of mental illness is a conditioner of the police officers' attitudes toward a person with mental illness subject to police transportation. Police officers seek a low profile approach, avoiding using instruments of authority (such as their police uniform, the police vehicle cage or handcuffs), in order to respect the patient's privacy.

#### The Presence of the Police Is What Counts

Police officers recognize that simply their presence, without any active control measure, has a positive impact on the person's behavior. The police escort results in the patients having a more controlled, submissive and respectful behavior, which maintains everyone's safety.

#### The Instrumentalisation of the Police

Despite the core role of police officers in the compulsory admission process and their privileged access to the patient's social background, police officers emerge and perceive their importance only as a law's tool, where their function is to fulfill their task: escort the patient to the hospital.

### Family Is Core in the Compulsory Admission Process

Family plays an important role for police intervention in the compulsory admission process, as they provide detailed and present information about the patient. This information is very important for police officers when preparing for their interactions with the person who is unwell. Family members can also provide direct access to the physical space of the patient (Table 3).

**Table 3:** Family is core in the compulsory admission process quotes.

**Family is core in the compulsory admission process**	
Facilitate the access to the patient	“*When the patient lives alone we cannot, (…) if the situation does not require an immediate intervention, we cannot enter in the patient's house against their will because if they do not open the door, we cannot break the door just because we have a warrant. The warrant does not allow us that and that is clear.”* (police officer 10)
Source of information	“*We have to talk with a relative to inform us about their behavior, which habits they have, what time they leave home. This is for us to see when it is the best moment to do the transport, if they are aggressive, if they react well or not with our presence. All of this has to be done and analyzed*.” (police officer 05)
	“*In the moment when we get the warrant, we contact always some of their relatives to plan what is the best way to approach the person. If he [the familiar] thinks is best for the patient that the first contact is through him [the familiar], if he is aggressive or not, if he is cooperative or not (…) When it is the first time for us with that person to be transported, the first thing we do is to contact a relative to know about what type of person he is*.” (police officer 07)

#### Facilitate the Access to the Patient

The Portuguese legal framework on compulsory admission does not allow police officers to force the entrance on a patient's home. This means that the involvement of significant others of the patient are crucial to gain access to them, typically a relative. Without access to the patient's home, the police officers have to wait until the patient is on a public highway in order to transport them to the hospital.

#### Source of Information

The amount of information that is provided to the police officers about the patient is usually scant. Patient's family or friends are a rich source of information about patient's personality, emotional state on that day, and informs their decision about when best to execute the warrant.

The involvement of family in the compulsory admission process is crucial to reach the person in a more effective manner, and to reduce the risk of any injuries or the need for physical force.

### Triad of Success

Transporting someone for compulsory admission is a challenging process given the complex symptoms exhibited by these patients and their social situation. Police officers are faced with unknown circumstances, and so adopt strategies to minimize the risks to themselves and to ensure an effective intervention (Table 4).

**Table 4:** Triad of success quotes.

**Triad of success**	
Obtain knowledge about the patient	“*We cannot go with our ‘eyes closed,' like we say. (…) We have to know more about the person's habits, if he is aggressive or not, or if he has the tendency to become aggressive, through the neighbors or somebody close to them. This is important for our service to occur under the normality and we can transport the patient without big problems”* (police officer 05)
Building a relationship	“*Is very important for us to get the patient's trust, this is very important for our intervention. I believe that it is the most important thing, to have the patient to trust us. If they do not believe us, it will be very difficult for us to do our service without taking some authority measures”* (police officer 05)
To be patient and not to rush	“*I seat myself on his table and I was pretending that I was eating and then I said ‘Hi John we have to go, you have an appointment at the hospital,' and he said ‘mister officer I am eating my codfish, please let me finish it' … You know, I was there for two hours pretending I was eating, but in the end he came with us with no trouble”* (police officer 05)
	“*I always want that the person to be transported comes with me through their free will. Sometimes it could take me fifteen, twenty minutes or half an hour, but I will talk with him until he understands that he has to come with me*” (police officer 09).

#### Obtain Knowledge About the Patient

The knowledge that they obtain about the patient is very important for the success of their intervention. This includes gathering information about the patient's behavior, habits, people that they live with and factors that will shape the way that the person will react to their approach. Police officers often obtain information from family members, however some patients live alone or do not have family. In these occasions, police officers have to use all the other resources available to get some insight into the person.

#### Building a Relationship

Since the first moment that police officers approach the patient, their objective is to build a rapport with the patient. This relationship building process and sense of patient's trust in the police officer is very time consuming. Nevertheless, for police officers this is a crucial factor in their intervention, and without it the trust in their approach can be compromised.

#### To Be Patient and Do Not Rush

The complex layout of the police activity under compulsory admission tasks requires a lot of sensitivity, patience and good communication skills from police officers. This police intervention is the most time consuming operation for Portuguese police officers who often are rushed in to solve challenging situations. Yet, in these cases, patience and dialogue are their only tool and can help them come across more as peers, which differs to their usual demeanor in the other types of police work they do. Establishing a conversation with a person with a serious mental disorder can be challenging, but also gives the police a sense of control over the situation. This is their strategy to understand the patient and gain their trust, allowing them to co-operate and accept that they need to leave with the police. This makes police officers more confident about dealing with the process.

### Views of Mental Illness

Police perceptions and beliefs about mental illness and about people with serious mental disorders facilitate better understanding of the patients' behavior and attitudes (Table 5).

**Table 5:** Views of mental illness quotes.

**Views of mental illness**	
Cyclic	“*The majority of the cases that we have are known to us, therefore we already took them more than once, and therefore we already know the person”* (police officer 07)
	“*I had an old man, who even died last year. I took him several times because he was already a regular person [that we transport]. He took the medication for a while and during that time he was ok, but then he used to drink alcohol, relapsed, and did not go to his periodic medical appointments. Then his medical doctor informed the public ministry, requesting a new order of transportation. Therefore he was out there”* (police officer 04)
Beliefs about people with mental illness	“*Mental illness is the illness of the century. The lack of awareness of this situation is dangerous because people are unconscious that they are unwell and that they need a doctor”* (police officer 03)
	“*A patient who has relapsed can be dangerous for themselves, or they can try to finish their life or to hurt others*” (police officer 04)
	“*If a person is unwell, he will end up becoming a dangerous to them or those around them”* (police officer 07)

#### Cyclic

Mental illness is perceived by police officers as cyclic, as they often transport the same patients in different periods (or relapses) of their illness. This cycle, characteristic of compulsory admissions, means police officers become an active agent of patient's treatment, although in a reactionary form during a crisis.

#### Beliefs About People With Mental Illness

Portuguese police officers see people with mental disorders as unpredictable, dangerous and with lack of insight about their illness and the need of treatment.

### Improving the Reality of Compulsory Admission

Police officers take responsibility for the patients' transportation until they reach the nearest psychiatric emergency ward for medical evaluation. Once there, police officers are responsible for the patient's deprivation of freedom and restitution (if required). This means that police officers have to remain with these patients and be present in almost all stages of a patient's compulsory admission. Therefore, police officers are able to provide a valuable insight into what could be improved in this process (Table 6).

**Table 6:** Improving the reality of compulsory admission quotes.

**Improving the reality of compulsory admission**	
Mismatch between safety and health	“*I have a warrant and I have to go to the line, and sometimes the line is huge to give the patients registration, and I have to wait. They say to me that is a medical emergency facility, and like me they have others. The difference is, in my case, other resources are being used*.” (police officer 01)
	“*The presence of the police there [in the hospital] is exhausting, even for the patient. They look at us and they feel that we are depriving them from their well-being. It ends up being too much time”* (police officer 09)
	“*We are talking about one police officer, by the way two police officers that remain in the hospital for six, seven hours waiting for a medical evaluation. In other words I believe that this is spending our resources”* (police officers 02)
	“*[In the hospital under compulsory admission order] we should have some priority because the patient is being transported under judicial order or by other entity. [Our patients' observation] should be a priority because other persons are involved and they have to be free of that duty”* (police officer 09)
Negative aspects resulting from the police intervention	“*The others will think ‘another thug.' If you are in a hospital and see uniformed a police officer (…) the others will judge.”* (police officer 01)
	“*Just the fact that we will be there does not mean the person has committed a crime. But in our society, if a common civilian is accompanied by a police officer, is immediately labeled as a criminal”* (police officer 02).
Police officers difficulties	“* Just as I had said before, my main difficulty is to convince the patient to come with us (…) just the fact that person has a mental illness is an obstacle difficult to overcome, because you cannot reach the communication, a healthy and coherent dialogue”* (police officer 03)
	“*Obviously that I am feeling limited. You have to have powerful argumentation skills to counteract certain kind of people, It is not easy, I am not a sales man (…) some situations that are not easy to deal and I am not prepared to deal with them*”. (police officer 09)
	“*Is the day by day experience. Today I do, tomorrow I will do and we will learn how to do according to the situation fits best for each scenario”* (police officer 05)
Try other solutions	“*I ask myself, ‘what if, instead of being the police taking care of the patient's transportation to the hospital, other professionals went to the patient's residence and ask for the collaboration of the police.' Would this not be more comfortable for the patient?”* (police officer 05)
	“*We should have some specialized physician working with us in these cases. Because for us it is complicated as we do not understand”* (police officer 06)
	“*In the warrant says ‘transportation warrant,' the language is clear, and then says ‘transport the citizen X to the psychiatric emergency ward Y.' (…) They should create a room, similarly as we transport a person in the cases of the victims of domestic violence to a separate room in our police departments. What is the problem? Because in that moment they are deprived of their freedom, and they will remain deprived until medical decision”* (police officer 01)
	“*We do not understand, when we have a hospital with all conditions, from private security, police officer assigned to the hospital, several rooms, and they prevent a police officer from doing other police duties. We are working to support the community and we wait there [at the hospital] for hours, waiting that somebody release us from being there to escort one patient, one person” (*police officer 09*)*

#### Mismatch Between Safety and Health

The controversy of compulsory admission for police officers is the shift from providing security to medical support. The hospital interaction is perceived by police officers as resource and time consuming. They strongly felt that patients under compulsory admission should have priority over other patients because they are there under a judicial order. The presence of the police in the hospital is perceived by the police officers to have a negative impact on the patient, being an expenditure of police resources, with many experiencing it like being detained.

#### Negative Aspects Resulting From the Police Intervention

Despite the care that police officers have when dealing with a person with mental illness, using a powerful institution which deals with the criminal world (i.e., the police), could have a negative impact upon the person that is being transported for medical evaluation.

#### Police Officers Difficulties

The interaction between police officers and people with mental disorders involves a special dynamic. Police officers have to transport the patient to the nearest psychiatric emergency service without any incident, which involves the person following their orders ideally in their own free will. The communication process is not easy, and police officers feel poorly prepared to do this.

These feelings of poor preparation can result from police officers' misunderstanding and/or lack of training on the behavior of people with mental illness. On the other hand, compulsory admission is not the core of police activity and the numbers of such admissions are low. Police officers learn how to deal with these patients from their own experiences.

#### Try Other Solutions

Police officers give suggestions to improve the compulsory admission process, which does not involve excluding police officers from the equation or discharging their responsibilities as police. They instead focus on patients' care improving their access to the mental health system. Their suggestions especially focused on police officers presence in hospital, which they express indignation with as it is a misuse of their resources and impacts their wider police duties.

## Discussion

### Key Findings

This study has raised awareness to the perceptions of police officers in dealing with compulsory treatment. Police officers felt confident about using Mental Health Law to approach these patients, which empowered them to help manage patients' behavior. However, they felt unprepared when dealing with the patients' behavior when this became challenging for them. Furthermore, police officers perceived patients as people who are unwell and require treatment. Police officers expressed that having an empathic approach, keeping a low profile, having a respectful interaction (e.g., using dialogue, patience and information about the person) was their main strategy. This demonstrates that police officers take special care when transporting patients against their will. Although they understand how mental illness can influence a person's cognition and behavior, they showed their own perceptions about mental illness and such patients (perceived as unpredictable, dangerous and without the capacity to judge their health status). Finally, our findings highlight their desire for increased support from other mental health professionals, for the process to be more efficient, and use less police resources. This would allow a balance between security and medical assistance, prioritizing the latter.

### Strengths and Limitations

To our knowledge this is the first study in Portugal that has focused on police officers and their interactions with people with serious mental disorders under compulsory admission, and therefore these findings add to a very limited literature base and hopefully set grounds for further work. The setting of this study is another strength as it included a wide range of police departments and police officers that perform compulsory admissions in Porto, including police officers with vast experience in this field. The geographical coverage of the study is also a strength, since different geographical areas may represent different social frameworks and provides us with rich and detailed information about the phenomena.

The study had however some limitations. Firstly, the sample of police officers interviewed was relatively small and did not allows us to explore and compare the differences in police officers views toward compulsory treatment, and whether this varied on the number of compulsory treatments they had previously carried out or their personal characteristics. Secondly, the sample targeted Porto's police departments and there is a possibility that in other areas of Portugal, with different characteristics, the experience of police officers may be different. Finally, our study only involved PSP (Policia de Segurança Pública) police officers. Other police officers from different police forces, like GNR (Guarda Nacional Republicana) who receive military training, may experience the compulsory admission process in a different way.

### Comparison With the Literature

In our study, police officers in Portugal expressed special care and understanding of patient's medical needs, promoting a collaborative relationship in what is an asymmetrical power relationship by nature, using tools, such as dialogue, patience, building a positive rapport and gathering information about the patient beforehand. This emotional bond with a person with a mental disorder has also been shown in research in other countries, such as with police officers in the United Kingdom (UK), where they express the desire to help and to have empathy and understanding of people with mental disorders (25). A gentle communicative style is required to gain trust, rapport and compliance from the person under compulsory admission (37). The strategy of using dialogue and giving time to patients to explain themselves is perceived by people with a serious mental disorder as a more humane treatment (38, 39). For patients, being treated as humans is crucial to perceive the interaction as positive and for that, factors, such as communication style, being listened to, gentle handling, putting them at ease and not rushing things are key (37, 39). This procedural justice activity framework is crucial for the patient's cooperation and influences perceptions of police legitimacy. When the police intervention is grounded in a procedural justice approach, the quality of police-citizens interaction is enhanced (26) and people voluntarily comply with the police (8). Procedural fairness may assume an important role in maintaining the person's identity in the community, enhancing the person's value, and may facilitate their involvement in the treatment with future repercussions in treatment adherence (8).

Furthermore, our results in this study show that the involvement of the family in this process has emerged as a core feature in this police intervention, given that it is a useful source of information about the patient and a relevant link and resource in the process. Certainly, family and social support is a cornerstone of the Portuguese mental health care, especially in the case of people with serious mental disorders (40). This is in line with the findings from previous research, which had already highlighted the importance of family, as having a central role in recovery. Its involvement is seen as a valuable and qualified resource, responsible for assisting with the care provision (41). Importantly, the way in which a family starts a compulsory admission process has an effect on how individuals consider the first days of admission and is decisive in the patient's whole experience (42). It has been emphasized already that working in partnership and having discussions between the staff and the family members contributes to the development of best practice (43), to deliver safe and effective interventions (41). Despite the importance of family in mental health care, some families in London reported that they are treated as an irrelevant and troublesome part by the British mental health system (44).

In Portugal, seeking further information about the patients and the family involvement in the process is current practice in Portuguese police compulsory admission interventions and seems to be one strategy to a successful intervention. From a patient' perspective, it is also important that police officers have access to some personal information prior to their arrival to the scene, in order to know how to handle the person, how to communicate and to keep the situation under control. Yet, this amount of information should be properly handled by appropriately trained police (45). Without this, patients could be put at a high level of risk (38).

Our findings indicate that police officers in Portugal acknowledged that their presence has a positive impact on the person's behavior, resulting in a more controlled, submissive and respectful attitude. On the other hand, some police officers perceived that their presence could have a negative impact upon the person that is being transported for medical evaluation.

Hospital admission represents a significant event in the lives of people with serious mental disorders (3). It is reported by many as a traumatic process, and signifies a loss of patient's autonomy (42). However, “objective” coercive measures do not seem to be related with reduced satisfaction in people who are deprived of their freedom to receive medical treatment (46), but “perceived” coercion seems to be (47). This suggests that measures engaged to promote patient's self-control, such as providing explanations and involving them in the decision-making (42), may decrease the level of perceived coercion in the process of compulsory admission, and may increase the patient's satisfaction with their care and the treatment overall (46). Improving patients' satisfaction with care during the compulsory admission process is important not only to provide the highest quality and humane service to patients who require care, but also because low satisfaction levels are linked with poor engagement with services and repeated involuntary admissions (48, 49).

Importantly, the literature reveals that to be in police custody can be experienced as shocking, humiliating, intense or forceful, especially if the patient was not violent, and even if the police officers were kind and gentle during the process (37). In fact, a long period of time in custody, especially in suicidal patients, might increase their mental distress (44). Police officers in the UK acknowledge that a person with mental health problems may be traumatized by the police intervention and that, as police officers, they were frequently perceived as a threat by such patients (25). Regardless of this, police officers did not want to cause or prolong distress for these people, but felt they were frequently placed in situations where they were the only service available to help people in crisis (25). This is perceived by police officers (25) and patients (50) as a consequence of the failure of health services, and the fact that they are not mental health specialists (51).

The idea that police officers should not be responsible for mental health patients in crises is shared by police officers in Greece (16), and in the UK (25). Particularly in the UK they emphasize that they are put far beyond the initial crises stage and that police and medical professionals should work together to get a positive outcome, instead of unnecessary arrests (25). Police officers reported dissatisfaction with the process of getting a person into a treatment facility (52), due to concerns about police time and resources consumption (16, 53). Feeling unsupported is not only about lack of medical support or about the services provided, it is also about the background information about the person, and the feedback on the situation (53).

Although Portuguese police officers feel that the health system does not respond appropriately to the police force and to the patients' requirements, they express disappointment especially for the negative consequences this has on police officers wider duties and patients distress.

### Implications of the Findings for Practice, Polices, and Research

Our study shows that interventions for police officers are still necessary in order to improve the compulsory admission process, such as: (i) providing adequate formal training and education on mental health that can help change the police misperceptions about people with serious mental illness, preparing the police officers to deal with patients' challenging behavior; (ii) improve communication and cooperation between the police, the medical community and social service providers, considering alternatives to improve their work and most importantly patients' care; and (iii) improve the conditions at the hospital emergency departments, so that patients referred compulsorily, are assessed as soon as possible.

Police officers expressed in this study that the existence of a law can give them legitimacy to deprive a person's freedom, although this is not a detention. This legal difference is well-recognized by police officers and is enforced in their interventions. Consequently, the police approach a person with mental illness by explaining that their function is not to arrest them, but instead to take them to a hospital for medical evaluation with an order determined by law. In this way the legitimacy of the intervention is legally guaranteed and police officers remain neutral in the decision-making process of compulsory admission. This has an important role in the patients' behavior and in the police confidence in managing this. In fact, the impact of the legislation in the patients' behavior and their perceptions of compulsory admission should be further studied. It seems that patients awareness of the legal order and of police officers responsibilities (i.e., once the patients realize that it is a legal order and that the police are just doing their job), they tend to improve cooperation and their experience (39).

Using the police to transport mentally unwell people in crisis represents a major expenditure of police resources, occasionally with some negative impact upon the patients, given their lack of preparation and the lack of support from mental health professionals. Therefore, it is important to sensitize police officers to the importance of their task and actively engage them to collaborate with mental health professionals. In general, police officers share the belief that it is not their responsibility to deal with mentally ill people in crises. It is imperative to change this to guarantee a more effective response in the compulsory admission process and a faster access of patients to the mental health services. A more efficient approach would reduce the time that police officers are away from their other usual duties, since the time that they spend during this task was their biggest complaint. The creation of joint task forces between police and mental health professionals could be highly beneficial, responding faster and reducing stigma/discrimination (51). A multi-agency strategy approach could also enhance the legitimacy of the intervention and contribute to the procedural fairness of the compulsory admission process (26). This suggests the need to further study the effectiveness of different approaches. Possibly that could lead to a change in the Portuguese mental health legal framework, where frontline medical provision is supported by police officers, if required, and social services. Future qualitative research with larger samples of police officers, and from different police forces, could further explore and compare the differences in their views toward compulsory treatment, and whether this is affected by the number of compulsory treatments they had done, by their background and/or personal characteristics.

Finally, our findings show that the police officers in Portugal approach compulsory admission as a help call, with full understanding that there is a person who needs medical attention and has to be treated with empathy, dignity and respect. They seek a humane interaction using all the means at their disposal to do the transport with minimal negative effects on them, and without use of force. Creating a rapport and trust are highlighted as crucial to enhance a relationship with a patient. For this, Portuguese police officers use dialogue as a strategy, but this can be too time consuming and a challenging process which they can experience as a major difficulty. For a better interaction, formal and standardized training and educational programs should be implemented to help increase the confidence levels of police officers during their interactions with people with mental disorders (52). Although they express that they rarely have to use force in these processes, Portuguese police officers perceive people with serious mental disorders as unpredictable, dangerous and without the capacity to evaluate their mental state. Their perceptions about mental illness may also result from their lack of knowledge and experience. The way police officers perceive people with mental illness is of major concern since insufficient mental health education could put people with serious mental disorders at risk (38), and enhance the chances of physical confrontation, as it may result in a situation where both parties misunderstand each other (14). Therefore, it is vital to further assert in the difficulties, concerns and beliefs of police officers across the country and beyond, and complement this research with further studies focusing on patients' perceptions about this police intervention. This input from different sectors is imperative to the development of formal and standardized education and training programs that would help police officers: (i) reducing the use of measures that may increase perceived coercion; (ii) changing their misperceptions about people with serious mental disorders; and (iii) helping them to determine the best strategy to provide the best care.

## Conclusion

This study illustrates Portuguese police officers experiences and perceptions during the process of compulsory admission, providing more information about this understudied area. It also highlights their efforts for a humane and empathic treatment that they provide when transporting people for compulsory admissions. Some aspects of the police intervention should be further developed for an improved and swifter process when transporting patients, and to promote a more humane and effective approach.

## Ethics Statement

Ethical approval for the study was obtained from the Ethics Committee (203/2017) of the Institute of Biomedical Sciences Abel Salazar (ICBAS) of the University of Porto and a favorable authorization was obtained from the National Formation Department of PSP (3F05-2017), prior to the interviews. Invited participants received information about the purpose of the study and the research method verbally and through information sheets prior to the interview. The contact details of the researcher were given to the interviewees for any further questions prior or after the interview. They were also informed about their right to withdraw their consent and withdraw from the study without any penalties and that confidentiality would be maintained. Written informed consent was obtained from participants prior to the interviews.

## Author Contributions

RS and MPC conceived the study. RS made all the contacts with the police, performed the interviews and led the analytic process. RS and MPC analyzed the results and wrote the paper.

### Conflict of Interest Statement

The authors declare that the research was conducted in the absence of any commercial or financial relationships that could be construed as a potential conflict of interest.
