# Supplementary material for: Experiences and Perceptions of Police Officers Concerning Their Interactions With People With Serious Mental Disorders for Compulsory Treatment
Source: Front Psychiatry. 2019 Apr 18;10:187. doi: 10.3389/fpsyt.2019.00187 (PMC6482210; doi:10.3389/fpsyt.2019.00187)
Supplement: Supplementary file 1 [file Data_Sheet_1.PDF]

U.PORTO
INSTITUTO DE CIÊNCIAS BIOMÉDICAS ABEL SALAZAR
UNIVERSIDADE DO PORTO

Participant number:

Socio-demographics

Gender: M ☐ F ☐

Age:

Ethnic background:

Education:

Rank:

Years of service in the Police:

Diagnosis of mental illness: Yes ☐ No ☐

(If yes, which?):

Frequency of compulsory admissions (transports/month):

Participation in any kind of mental health education program: Yes ☐ No ☐

(If yes, what was the main goal of it?):
