# Supplementary material for: Experiences and Perceptions of Police Officers Concerning Their Interactions With People With Serious Mental Disorders for Compulsory Treatment
Source: Front Psychiatry. 2019 Apr 18;10:187. doi: 10.3389/fpsyt.2019.00187 (PMC6482210; doi:10.3389/fpsyt.2019.00187)
Supplement: Supplementary file 2 [file Data_Sheet_2.PDF]

U.PORTO
INSTITUTO DE CIÊNCIAS BIOMÉDICAS ABEL SALAZAR
UNIVERSIDADE DO PORTO

Interview Topic Guide

Experiences and perceptions of police officers concerning their interactions with people with serious mental disorders for compulsory treatment

The following questions are intended as a guide for the interviewer in conducting semi-structured interviews. It is not expected that the interviewer will ask each question in exactly the words stated here, and the interviewer will be encouraged to follow up on areas that they seem to have particular interest, enthusiasm or concern with.

1. **In your view, what is the main goal of a mental health law?**
   Probe about benefits and risks of using it
   Probe about contribution to mental health promotion
   Probe about contribution to the security of the community
2. **In your view, what should be the role of the police force in the mental health system, if any?**
   Probe about its importance
   Probe about the circumstances in which police forces should operate
3. **In your experience, how do you feel when you interact with a person with a mental disorder?**
4. **In your experience, which measures do you adopt to transport a patient for a compulsory admission assessment?**
   Probe about utilization of the uniform
   Probe about getting information about the patient
   Probe about scheduling the escort
5. **What are the patient's reactions when they see you?**
   Probe about the police officer's reaction
   Probe about the content of what patients say to the police officers
   Probe about how police officers feel of patient's response to them
6. **Which strategies do you adopt when interacting with the patient?**
   Probe about the way they speak to the patient
   Probe about how these strategies help with dealing with the patient in a peaceful way
7. **What challenges do you encounter when you transport a person for the compulsory admission procedure?**
   Probe about how these are overcome
   Probe about the procedures adopted when the patient refuses to go with the police officer
   Probe about how they deal when the patient's behaviour escalates to a more aggressive behaviour
   Probe about the consequences of police officers' actions in the patient's behaviour
8. **What do you think about the obligation of police officers to remain in the hospital during the patient's medical evaluation?**
   Probe about whether their presence affects the patient's behaviour
   Probe about whether their presence (next to the patient) has an impact in other people's perceptions of that patient
9. **Do you have any further information that you want to add?**
